# *Moringa oleifera* Protects SH-SY5YCells from DEHP-Induced Endoplasmic Reticulum Stress and Apoptosis

**DOI:** 10.3390/antiox10040532

**Published:** 2021-03-29

**Authors:** Ines Amara, Maria Laura Ontario, Maria Scuto, Gianluigi Maria Lo Dico, Sebastiano Sciuto, Valentina Greco, Salwa Abid-Essefi, Anna Signorile, Angela Trovato Salinaro, Vittorio Calabrese

**Affiliations:** 1 Department of Biomedical and Biotechnological Sciences, University of Catania, Torre Biologica, Via Santa Sofia 97, 95125 Catania, Italy; ines.amara15@yahoo.fr (I.A.); marialaura.ontario@ontariosrl.it (M.L.O.); mary-amir@hotmail.it (M.S.); gigilodico@gmail.com (G.M.L.D.); ssciuto@unict.it (S.S.); vgreco@unict.it (V.G.); calabres@unict.it (V.C.); 2 Laboratory for Research on Biologically Compatible Compounds, Faculty of Dental Medicine, University of Monastir, Rue Avicenne, Monastir 5019, Tunisia; salwaabid@yahoo.fr; 3 Pathology Unit, Centro di Riferimento Oncologico di Aviano (CRO) IRCCS, 33081 Aviano, Italy; 4 Department of Basic Medical Sciences, Neurosciences and Sense Organs, University of Bari, Piazza G. Cesare, 11, 70124 Bari, Italy

**Keywords:** di-(2-ethylhexyl) phthalate, *Moringa oleifera*, endoplasmic reticulum stress, vitagenes, oxidative stress, apoptosis, mitochondrial respiratory complexes

## Abstract

*Moringa oleifera* (MO) is a medicinal plant that has been shown to possess antioxidant, anticarcinogenic and antibiotic activities. In a rat model, MO extract (MOe) has been shown to have a protective effect against brain damage and memory decline. As an extending study, here, we have examined the protective effect of MOe against oxidative stress and apoptosis caused in human neuroblastome (SH-SY5Y) cells by di-(2-ethylhexyl) phthalate (DEHP), a plasticizer known to induce neurotoxicity. Our data show that MOe prevents oxidative damage by lowering reactive oxygen species (ROS) formation, restoring mitochondrial respiratory chain complex activities, and, in addition, by modulating the expression of vitagenes, i.e., antioxidant proteins Nrf2 and HO-1. Moreover, MOe prevented neuronal damage by partly inhibiting endoplasmic reticulum (ER) stress response, as indicated by decreased expression of CCAAT-enhancer-binding protein homologous protein (CHOP) and Glucose-regulated protein 78 (GRP78) proteins. MOe also protected SH-SY5Y cells from DEHP-induced apoptosis, preserving mitochondrial membrane permeability and caspase-3 activation. Our findings provide insight into understanding of molecular mechanisms involved in neuroprotective effects by MOe against DEHP damage.

## 1. Introduction

Di-(2-ethylhexyl) phthalate (DEHP) belongs to the class of phthalates, which are widely employed as plasticizers to impart elasticity to polymer products [1]. This property is at the origin of the variety of its applications: in the domestic field (wall coverings, leather goods), food (food and beverage packaging), medical (infusion bags, tubing devices), and cosmetic [2]. Human exposure to DEHP may arise via food, air, or medical devices, as this phthalate is easily absorbed through all exposure routes. Various adverse effects have been associated with DEHP exposure, namely neurotoxicity, reprotoxicity, nephrotoxicity, hepatotoxicity, and endocrine disruption [3,4,5,6]. DEHP-induced neurotoxicity involves several cellular mechanisms such as oxidative stress, nuclear factor erythroid 2-related factor (Nrf2) pathway, mitochondrial dysfunctions, and apoptosis [7,8]. Moreover, it has been reported that, at particular concentrations, DEHP can exert neurotoxic effects following both prenatal and postnatal exposures. Specifically, in rats, prenatal exposure to DEHP disrupts central nervous system development and decreases brain weight [9]. Also, it has been proven that DEHP poses a negative impact on hippocampal development in male rats [10]. Postnatal DEHP exposure is known to cause motor hyperactivity and significantly decreases the number of midbrain dopaminergic neurons [11]. In humans, association between autism spectrum disorders and phthalate exposure has been found [12]. Due to these cellular disturbances and their widedistribution, phthalates attract public concern. In order to limit the toxicity of DEHP, the increase in the consumption of diet components such as vitamins and antioxidants could be considered. Therefore, studies on the effect of antioxidants, particularly those consumed in foods, areconsidered to be of great interest in preventing DEHP-induced cell damage. For centuries, traditional medicine has used medicinal plants which are rich in secondary metabolites, with important biological activities, and phytochemicals used in drugs manufacturing [13]. *Moringa oleifera* (MO), belonging to the Moringaceae family, is an important source of nutrients because of its high content of bioactive ingredients present in the leaves, seeds and pods of the plant. In fact, MO provides protein, calcium, potassium, iron, vitamin A, and vitamin C [14]. MO appears to possess various health-promoting properties. The flavonoids and polyphenols contained in MO confer neuroprotective, antioxidant, antibacterial, and anti-inflammatory properties [15,16,17].It has been demonstrated that MO extract (MOe) exerts protective effects on many organs, including liver, reproductive system, and central nervous system [18,19,20]. The use of MOe at different concentrations and incubation times makes this compound very versatile and itcan also have antiproliferative/anticancer activity [21]. The potential anticancer effect of MOe has also been investigated, in vivo, where MO extract administration was able to reduce the tumor weight and the volume of tumor-bearing mice, while increasing the lifespan [22], as well as in vitro in cancerous human alveolar epithelial cells [23]. Antiproliferative activity has been reported for isolated bioactive compound and/or enriched extracts from MOe. In a model of lead-induced liver damage and inflammation, MOe prevented oxidative stress, an affect associated with decreased nuclear factor kappa-light-chain-enhancer of activated B cells NF-κB levels, inflammation, and apoptosis [24]. Increasing evidence reports that MO activates phase 2 response, resulting in expression of the Nrf2/heme oxygenase 1 (HO-1) antioxidant pathway [25]. Interestingly, Nrf2 (nuclear factor erythroid 2-related factor) represents an essential defensive mechanism against oxidative stress and mitochondrial dysfunction [26,27]. Nrf2, under physiological conditions, is localized in the cytosol and kept inactivated by binding to its inhibitor Kelch-like ECH-associated protein 1 (Keap1). Exposure to DEHP induces cytotoxicity associated with reactive oxygen species (ROS) generation, leading to Nrf2 translocation and accumulation into the nucleus where, by binding to the antioxidant response element (ARE), it promotes transcription of multiple target genes, which include phase II detoxification enzymes, NAD(P)H:quinone oxidoreductase 1 (NQO1), heme oxygenase 1 (HO-1), and glutathione S-transferase (GST) P, Pi, and A1 isoforms [28,29,30,31]. In addition, the Nrf2 promoter also contains ARE sequences, making its transcript level also a marker of Nrf2 pathway status. In line with these observations, Nrf2 encodes vitagene antioxidant pathways able to counteract different forms of stress (e.g., oxidative, environmental, and endoplasmic reticulum (ER) stress). The latter involves redox-sensitive genes, such as heme oxygenase-1 (HO-1), heat shock proteins (Hsps), thioredoxin, and the sirtuinsystem, termed vitagenes [32,33,34]. Notably, recent evidence suggested that polyphenols from plant and fungal species activate the Nrf2/HO-1 pathway, conferring cytoprotection against ROS generation in a hormetic-like manner [35,36,37,38,39,40,41,42]. HO-1 catalyzes the conversion of heme to biliverdin, ferrous iron, and carbon monoxide, which can directly scavenge free radicals. Interestingly, our recent in vivo and in vitro studies with mushrooms polyphenols have demonstrated their neuroprotective effects by modulation of stress-responsive vitagenes in different brain regions of rats [43,44,45], as well as in pheochromocytoma 12 (PC12) cells against DEHP-induced neurotoxicity [46]. Hence, the present research was designed to examine the efficacy of MOe in protecting human neuroblastome cells against toxic effects induced by DEHP. In particular, we have analyzed the effect of pretreatment of MO on DEHP-induced oxidative stress, mitochondrial dysfunctions, antioxidant protein deregulation, and apoptosis.

## 2. Materials and Methods

### 2.1. Chemicals and Reagents

Ammonium acetate, acetone, and 2-propanol and formic acid werepurchased from Carlo Erba (Milan, Italy). Standard solutions of vanillic acid, syringic acid and chlorogenic acid were purchased from Extrasynthese (Genay Cedex, France). Acetonitrile and methanol were purchased from Merck (Darmstadt, Germany). The solid-phase extraction SPE cartridges used were Waters Oasis Hydrophilic-Lipophilic Balance (HLB) (200 mg) and ultrapure deionized water, with resistivity of 18.2 MΩ cm, obtained from the Milli-Q^®^ Integral water purification system with Q-pod (Millipore, Bedford, MA, USA). All solutions were filtered through 0.45 membranes (Millipore, Bedford, MA, USA) and degassed before use. Ferulic acid, gallic acid, kaempferol, quercetin, and rutin, N,O-bis(trimethylsilyl)trifluoroacetamide plus 1% of trimethylchlorosilane, Di(2-ethylhexyl)phthalate, Dulbecco’s modified eagles Medium (DMEM) high glucose, 2′,7′-dichlorodihydrofluoresceine diacetate (DCFH2-DA), 3-4,5-dimethylthiazol-2-yl,2,5-diphenyltetrazolium bromide (MTT), phosphate buffer saline (PBS), fetal bovine serum (FBS), streptomycin and penicillin mixture, and trypsin-Ethylenediaminetetraacetic acid (EDTA) were acquired from Sigma-Aldrich S.r.l. (Milan, Italy).

### 2.2. Moringa Preparation, Extraction, and Analysis by Gas Chromatography—Tandem Mass Spectrometry (GC-MS/MS) and Liquid Chromatography—Tandem Mass Spectrometry (LC-MS/MS)

Moringa (*Moringa oleifera* Lamk.) sprouts powder was supplied by EDYNEA (Vicenza, Italy). Powder from MO was obtained according to the process described in the registered patent (WO/2020/021071), which relates to a process for the preparation of a raw material enriched with isothiocyanates, which are known for their anti-inflammatory properties, and polyphenols, which are known for their antioxidant action, starting from Moringa sprouts by soilless cultivation and/or form sprouts harvested from the adult plant. In brief, the preparation of Moringa sprouts from seeds comprises selection of seeds, germination, and growth under controlled soilless conditions, harvesting of aerial parts, seed, and root residues. The preparation of the powder from sprouts obtained by the process described above proceeds with washing of the aerial parts and extraction by a screw extractor to obtain a solid phase and a liquid phase, which are homogenized and then subjected to a freeze-drying process to yield, after grinding, the final powder preparation. Extraction of polyphenol compounds was accomplished by dissolving two 0.125 g aliquots of MO powder in 10 mL of methanol and 10 mL of acetonitrile respectively, in an orbital shaker incubated (Professional 3500 Orbital Shaker, VWR International Srl, Milan, Italy) at 5 °C for 24 h.The extracts were then filtered using a 0.45 μm syringe filter (Thermo Fisher Scientific Inc., Waltham, MA, USA).The filtrateswere then loaded onto the SPE cartridges, which had been pre-conditioned by treating them, in sequential order, with 4 mL of methanol and 2 mL of deionized water.After washing with 2 × 10 mL of water, compounds of interest were eluted with 1 mL of methanol for high-performance liquid chromatography − tandem mass spectrometry (HPLC-MS/MS) analysis and 1 mL of acetonitrile for GC-MS/MS analysis.

To perform HPLC-MS/MS analysis, a Q-Exactive Plus Hybrid Quadrupole-Orbitrap^TM^ Mass Spectrometer (Thermo Fisher Scientific, Waltham, MA, USA), equipped with a HESI (Heated ElectroSpray Ionization) source, was used.Analyses wererunin both positive and negative modes in order to determine the exact mass of each analyte. Mass spectrometer parameters were: sheath gas flow rate, 35 (arbitrary units); auxiliary gas flow rate, 10 (arbitrary units); spray voltage, 3.50 kV; capillary temperature, 300 °C; tube lens voltage, 55 V; heater temperature, 305 °C; scan mode: full scan; scan range (*m*/*z*) 100–1000; microscans, 1 *m*/*z*; positive resolution: 70,000; Fourier transform (FT) automatic gain control (AGC) target: 3 × 10^6^; negative resolution: 35,000; automatic gain control (AGC) target: 1 × 10^6^; maximum injection time (IT): 100 ms.

HPLC parameterswere the following: column temperature, 30 °C; sample temperature, 6 °C; flow rate, 0.2 mL min^−1^. The autosampler sample holder temperature was maintained at 7 °C. Mobile phases consisted of eluent A: 30 mM ammonium acetate (pH 5), eluent B: methanol, eluent C: 0.5% formic acid in water, and eluent D: acetonitrile/acetone/2-propanol (4:3:3, *v*/*v*/*v*). Mobile phases B, C, and D were required for the on-line cleanup of the analytes onTurboFlow^TM^, whilemobile phases A and B were used to perform the subsequent analytical separation. The sample injection volume was 5 μL.

The two on-line columns Cyclone P column (50 mm ×0.5 m, 60 μm particle size, 60 Å pore size, Thermo Fisher Scientific, Waltham, MA, USA) and Hypersil Gold (2.1 × 100 mm, 1.7 μm particle size) were eluted by the same gradient program according toLópez-Gutiérrezet al. and Lo Dico et al., with some modifications [45,47].The total run time was 10 min. Data analysis was performed using Thermo Scientific XCalibur (Thermo Fisher Scientific, Waltham, MA, USA) version 4.0 software and Qual Browser.

GC-MS/MS analyses were carried out using a Thermo Scientific TSQ-Quantum XLS Triple Quadrupole gas chromatography mass spectrometry system, equipped with a TR-5MS column 5% phenyl methyl siloxane, 30 m × 250 μm× 0.25 μm. Samples were trimethylsilyl (TMS)-derivatized prior to gas chromatography and 5μL of each samplewasinjected into a Programmed Temperature Vaporizer (PTV). MS acquisition time was 5–45.00 min in full scan mode [50–1000 atomic mass unit (amu)], with scan time 0.272, and Q1 peak width 0.70 eV in positive and negative polarity. Oven temperatures were: initial holding time of 1.00 min at 63 °C, rate of 5 °C/min up to 330 °C.

### 2.3. Cell Culture and Treatment

Human neuroblastome SH-SY5Y were maintained under their undifferentiated form and cultured in DMEM, 10% fetal bovine serum (FBS), 1% L-glutamine (200 mM), 1% of a mixture of penicillin (100 International Units (IU)/mL) and streptomycin (100 μg/mL), at 37 °C with 5% CO_2_. DEHP stock solution used during this study was 2 mM freshly prepared in pure ethanol before treatments [48]. For the latter, its concentration for treatments never exceeded 0.5%. The MO powder was dissolved in ultrapure water and formulated to 1 mg/mL. The solution was filtered through a 0.22 μm microporous membrane and diluted to the desired concentrations (50–400 µg/mL) in DMEM. Then, the MOe solutions were stored at 4 °C.

### 2.4. Cell Toxicity Assay (MTT Assay)

SH-SY5Y cells line were seeded on 96-well plates at a density of 25 × 10^3^ cells/well and treated for 24 h at 37 °C with different concentrations of DEHP alone, ranging from 5 to 200 µM or MO alone (10–100 µg/mL). Cells were also exposed to MOe (10, 50, 100, and 150 µg/mL) 24 h prior to DEHP treatment (55 µM). At the end of the designed incubation time, the culture medium was removed and replaced by 200 µL medium containing 0.5 mg/mL tetrazolium salt [3-(4,5-dimethylthiazol-2-yl)-2,5-diphenyltetrazolium bromide (MTT)] and incubated for 3 h at 37 °C. The MTT solution was then eliminated and 100 µL of DMSO was added to dissolve the converted purple dye in microplates. The absorbance was measured with a microplate reader spectrophotometer (BioTek, Elx800, Winooski, VT, USA) at a wavelength of 570 nm. Cell viability was expressed as percentage of formazan formation in treated samples as compared to control cells. The half maximal inhibitory concentration (IC50) values were defined as the concentration inducing a 50% loss of cell viability.

### 2.5. Reactive Oxygen Species Determination

The intracellular ROS levelwas measured by fluorometric assay using permeable 2′,7′-dichlorodihydrofluoresceine diacetate (DCFH2-DA). After diffusion inside the cell, the probe is hydrolyzed by intracellular esterases to non-fluorescent dichlorodihydrofluorescein (DCFH) and then oxidized to fluorescent DCF by ROS or reactive nitrogen species. SH-SY5Y cells were seeded on 24-well culture plates at 10^5^ cells/well for 24 h. Then, cells were incubated with DEHP (55 µM) alone or combined to MO (100 µg/mL) after 2 h pre-treatment. After 24 h incubation at 37 °C, cells were treated with 20 μM DCFH2-DA for 30 min. Finally, cells were washed with the phosphate-buffered saline (PBS) and intracellular production of ROS was measured by fluorometric detection of DCF oxidation on a fluorimeter (Biotek FL 800×) with an excitation wavelength of 485 nm and emission wavelength of 522 nm. The DCF fluorescence intensity is proportional to the intracellular ROS content.

### 2.6. Measurement of Mitochondrial Complex Activities

Complex I, II–III, IV and complex V activities were assayed in mitochondrial membrane-enriched fractions obtained from SH-SY5Y cells. The cells were trypsinized, washed with ice-cold PBS, frozen in liquid nitrogen, and kept at −80 °C until use. To prepare the mitochondrial membrane-enriched fractions, cell pellets were thawed at 2–4 °C, suspended in 1 mL of 10 mM Tris-HCl (pH 7.5), supplemented with 1 mg/mL BSA, and exposed to ultrasound energy for 15 s at 0 °C. The ultrasound-treated cells were centrifuged for 10 min at 600× *g*, 4 °C. The supernatant was centrifuged again for10 min at 14,000× *g*, 4 °C, and the mitochondrial membrane-enriched pellet was suspended in 0.1 mL of PBS. The NADH:ubiquinone oxidoreductase (complex I) activity was assayed, using 50 μg of proteins, in 40 mM potassium phosphate buffer, pH 7.4, 5 mM MgCl_2_, 3 mM KCN, 1 μg/mL antimycin, 200 μM decylubiquinone, following the oxidation of 100 μM NADH at 340–425 nm (Δε = 6.81 mM^−1^ cm^−1^). The activity was corrected for the residual activity measured in the presence of 1 μg/mL rotenone [49]. Succinate-cytochrome c oxidoreductase (complex II + III) activity was assayed in 25 mM potassium phosphate buffer, pH 7.4, 5 mM MgCl2, in the presence of 20 mM succinate, 2 mM of potassium cyanide (KCN), 65 µM decylubiquinone, and 20 µM cytochrome c, using 50 µg of protein. The cytochrome c reduction was followed at 550–540 nm (∆ε = 19.1 mM^−1^·cm^−1^). The activity of cytochrome c oxidase (complex IV) was measured following the ferrocytochrome c oxidation at 550–540 nm (Δε = 19.1 mM^–1^ cm^−1^). Complex V activity (Adenosine triphosphate (ATP) hydrolase activity) was measured using an ATP-regenerating system. 100 µg of protein were resuspended at 0.1 mg protein/mL in a buffer consisting of 375 mM sucrose, 75 mM KCl, 30 mM Tris-HCl pH 7.4, 3 mM MgCl_2_, 2 mM Phosphoenolpyruvate (PEP), 55 U/mL lactate dehydrogenase, 40 U/mL pyruvate kinase, 0.3 mM NADH. The reaction was started by the addition of 1 mM ATP and the oxidation of NADH was followed at 340 nm [50].

### 2.7. RNA isolation and qRT-PCR

Total RNA was isolated from cultured cells using RNeasy Mini Kit (Qiagen, Hilden, Germany) referring to the manufacturer’s instructions. An amount of 1 µg was reverse-transcribed in cDNA with Variable Input, Linear Output (VILO) SuperScript (Invitrogen, Monza, Italy). Real-time PCR experiments were performed in the StepOne Thermocycler (Applied Biosystems, Monza, Italy). cDNA was amplified using SYBR Green PCR Master Mix (Applied Biosystems, Monza, Italy). After an initial denaturation at 95 °C for 15 min, 40 cycles of amplification were performed under the following conditions: 94 °C for 15 s, 60 °C for 30 s, and 72 °C for 30 s. This experience was repeated 3 times. β-actin was performed to normalize for differences in RNA input. qRT-PCR primer sequences’ primers were obtained from Invitrogen: 5′ ACGGTGGAGTTCAATGAC 3′ (F) and 5′ TGTTGGCTGTGCTTTAGG 3′ (R) for Nrf2 (NM_031789.2); 5′ GAAGAGGAGATAGAGCGAAAC 3′ (F) and 5′ TGTGGCTGGTGTGTAAGG 3′ (R) for HO-1 (NM_012580.2); 5′-GCGCATGAAGGAGAAAGAAC-3′ (F) and 5′-CCAATTGTTCATGCTTGGTG-3′ (R) for CHOP (ENSG00000175197); 5′-CATTGGTGGCCGTTAAGAATGACCAG-3′ (F) and 5′-AGTATCGAGCGCGCCGTCGC-3′ (R) and 5′-CACGGCATTGTCACCAACT -3′ (F) and 5′-TCAGTCAGCAGCACAGGAT-3′ (R) for β-actin (NM_001033084.1). Standard curves and validation experiments were performed for each real-time PCR assay, allowing us to use the comparative Ct (2^−ΔΔCt^) method to calculate changes in gene expression.

### 2.8. Protein Extraction and Western Blotting

For protein extraction, the cells were lysed using the buffer containing N-2-hydroxyethylpiperazine-N-ethanesulfonic acid (HEPES) 0.5 M, 0.5% Nonidet-P40, 1 mM Phenylmethylsulfonyl fluoride (PMSF), 1 mg/mL aprotinin, and 2 mg/mL leupeptin, pH 7.4. Equal amounts of proteins were separated by 12% Sodium dodecyl sulfate (SDS)-polyacrylamide gel electrophoresis transferred onto nitrocellulosemembrane and then incubated for 1 h at room temperature in a blocking solution containing 20 mM Tris pH 7.4, 150 mM NaCl and Tween 20 (TBS-T), and 2% milk powder. Membranes were incubated overnight at 4 °C with appropriate primary antibodies: anti-Nrf2 polyclonal (SC-13032, Santa Cruz Biotech), anti-HO-1 (SC-10789, Santa Cruz Biotech Inc.), anti-GRP78 (SC-13539, Santa Cruz Biotech Inc., Dallas, TX, USA), or anti-CHOP (SC-7351, Santa Cruz Biotech Inc.). For protein loading controls, the same membrane was incubated with anti-β-actin (ab8227, Abcam, Cambridge, UK) for 1 h. Membranes were then incubated with the secondary polyclonal antibody conjugated with horseradish peroxidase for 1 h at room temperature. The membranes were then washed three times with TBS-T for 5 min. Finally, the membrane was incubated with the Super Signal chemiluminescent detection system kit (Cod34080 Pierce Chemical Co., Rockford, IL, USA) to detect protein bands corresponding to each antibody using Gel Logic 2200 PRO (Bioscience). The immunoreactive bands were analyzed with ImageJ software to determine the relative density (RD). The molecular weight of proteins analyzed was determined using protein molecular weight standards. The result is shown as a ratio between the intensity of the protein tested in the treated cells compared to that in the untreated cells.

### 2.9. Mitochondrial Membrane Potential (ΔΨm) Assay

Mitochondrial membrane potential is measured according to the cell membrane uptake of the cationic fluorescent dye rhodamine-123. SH-SY5Y cells (25 × 10^3^ cells/well in 96-well plates) were treated with DEHP (55 µM) alone or combined to MOe (100 µg/mL) for 24 h. Next, cells were incubated with rhodamine-123 for 15 min (37 °C, 5% CO_2_). The up-taken rhodamine-123 was detected by fluorimetric detection. The results were expressed as the percentage of rhodamine fluorescence absorbed by the treated cells from the fluorescence measured in the untreated cells.

### 2.10. Cell Death Induced by DEHP

Annexin V/propidium iodide (AnnV/PI) double staining was used to distinguish necrotic from apoptotic cells. The combination of PI and fluorescein isothiocyanate (FITC)-AnnV allow to distinguish between viable (AnnV−/PI−), early apoptotic (AnnV+/PI−), and late apoptotic/necrotic (AnnV+/PI+) cells. The Annexin V assay was accomplished based on the manufacturer’s instructions (Annexin V-FITC kit, Bender MedSystems, Wien, Austria). Fluorescence of at least 5000 cells was detected by flow cytometerFACSAria III (BD Biosciences, San Jose, CA, USA).

### 2.11. Caspase-3 Activity Assay

Caspase-3 activity was performed according to the manufacturer’s instructions (BD Pharmingen). At half confluence, SH-SY5Y cells were cultured in the presence of DEHP alone (55 µM), MOe alone (100 µg/mL), or DEHP with MOe at 37 °C for 24 h. After lysing cells for 30 min, they wereincubated for 1 h at 37 °C with the caspase-3-like proteases substrate Ac-DEVD-AMC (Ac-Asp-Glu-Val-Asp-7Amino-4-methylcoumarin). The aminomethylcoumarin (AMC) released was then quantified using a Perkin-Elmer fluorimeter at an excitation/emission wavelength of 380/460 nm. The bicinchoninic acid assay (Pierce, Rockford, IL, USA) was used to measure the total protein content in the lysates and then the results were expressed as the percentage of activity in lysates from control cultures.

### 2.12. Statistical Analysis

Each experiment was assessed three times separately and results were expressed as the mean ± standard deviation (SD) of the means. We have used theone-way analysis of variance (ANOVA) test to evaluate differences among groups, followed by Tukey’s post hoc test. We have used Student’s *t* test to compare differences between two groups. Differences were considered significant at *p* < 0.05.

## 3. Results

### 3.1. Analysis of MOe by LC/MS and GC/MS

**Figure 1:**
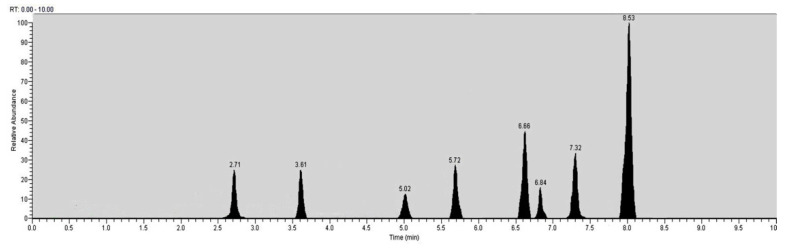
Representative Liquid Chromatography/MS(Orbitrap^TM^) chromatogram.

**Figure 2:**
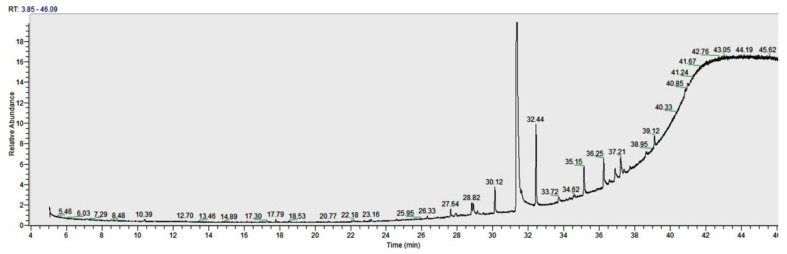
Representative Gas Chromatography—Tandem Mass Spectrometry (GC-MS/MS) chromatogram.

Liquid Chromatography/MS (Orbitrap^TM^) analysis (Figure 1) shows the largest relative abundance found as kaempferol (29%). Also relevant is the abundance of quercetin (15%), followed by ferulic acid (14%), gallic acid, and chlorogenic acid (11%). Rutin and vanillic acid were shown to be 3% and 4%, respectively. A derivatization with trimethysilane was carried out in order to improve the chromatography and the identification of the peaks by GC-MS/MS (Figure 2). Recognition of the analytes was conducted both with the analysis of single standards and with the method of exact masses and convolutions with algorithms. The relative abundance relative to the area of peaks recognized with certainty is shown in Table 1. Gallic acid with a relative abundance of 41%, quercetin 5%, and kaempferol 8% were detected. Other compounds found in GC are fatty acids, axatanthines, and sterols. The components present in our sample identified through LC/MS and GC/MS chromatographic analysis are shown (Figure 1 and Figure 2).

### 3.2. Effect of MOe on DEHP-Induced Cytotoxicity

**Figure 3:**
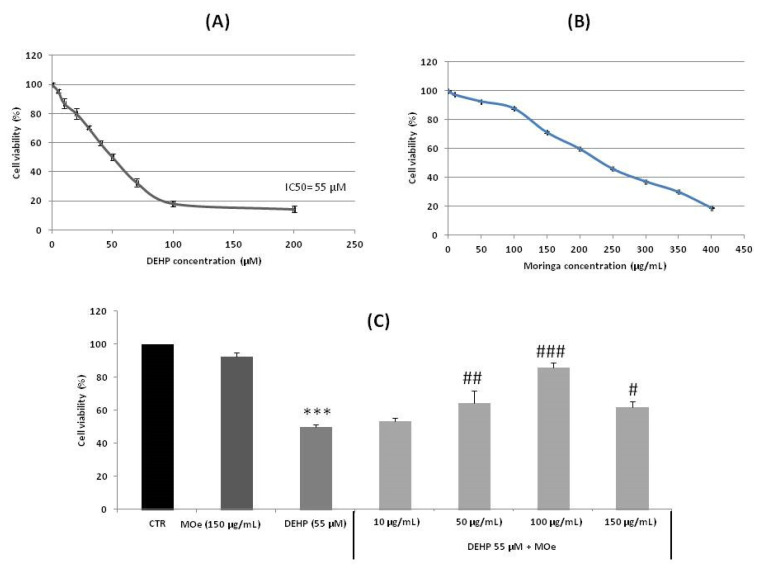
(**A**,**B**) Cytotoxic effect of di-(2-ethylhexyl) phthalate (DEHP) and *Moringa oleifera* extract (MOe) on SH-SY5Y cells. Cells were treated with DEHP or MOe at the indicated concentrations for 24 h. Cell viability was determined using the MTT assay and expressed as percentages of viability. (**C**) MOe reduces DEHP-induced cytotoxicity in SH-SY5Y. Cells were pretreated for 2 h with MO (10, 50, 100, and 150 µg/mL) before DEHP treatment for 24 h (55 µM). Data are expressed as the mean ± standard deviation (SD) of three independent experiments. *** p < 0.001 vs. control, *# p* < 0.05, *## p* < 0.01, and *### p* < 0.001 vs. DEHP alone.

DEHP treatment of SH-SY5Y cells with concentrations ranging from 5 to 200 μM caused a significant decrease in the cell viability after 24 h (Figure 3A). The IC50 value, assessed after 24 h of cell treatment, was around 55 µM. Moreover, MOe alone did not cause cytotoxic effects towards SH-SY5Y cells at selected low concentrations (Figure 3B), while pretreatment with MOe for 2 h at 10, 50, and 100 µg/mL significantly decreased DEHP-mediated cytotoxicity (Figure 3C). The high concentration tested of MOe (150 µg/mL) is not toxic towards SH-SY5Y cells, it has shown a viability ranging from 70% (Figure 3B) to 90% (Figure 3C).

### 3.3. Effect of MOe on DEHP-Induced ROS Generation

**Figure 4:**
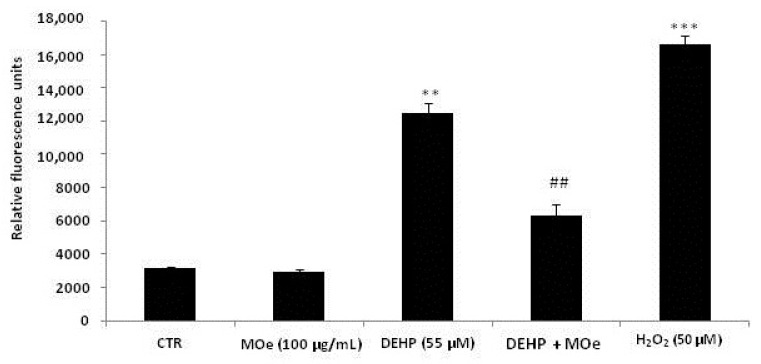
Effect of MOe on DEHP-induced reactive oxygen species (ROS) generation. SH-SY5Y cells were pretreated with MOe (100 µg/mL) for 2 h before DEHP treatment for 24 h (55 µM) or with H_2_O_2_ (20 μM) as a positive control. The relative intracellular ROS production was assessed by measuring the fluorescence of DCF, resulting from the oxidation of DCFH mainly by H_2_O_2_. Data are expressed as the mean ± SD of three separate experiments. ** p < 0.01 vs. control and *## p* < 0.01 vs. DEHP alone, *** p < 0.001 vs. control.

DEHP toxicity has been related to oxidative stress generation in numerous cell lines. Consequently, we test whether MOe exerts its suggested antioxidant property and modulates ROS level generated by the DEHP in human neuroblastoma cells. Hence, after DEHP treatment, intracellular ROS was measured in the absence or presence of 100 µg/mL of MOe. As show in Figure 4, DEHP treatment induced a marked ROS generation increase ofabout 2.8-folds with respect to the control group. Pre-treatment with MOe at 100 µg/mLwas able to reduce the intracellular ROS generated by DEHP in SH-SY5Y cells.

### 3.4. Effect of MOe on DEHP-Induced Mitochondrial Dysfunctions

**Figure 5:**
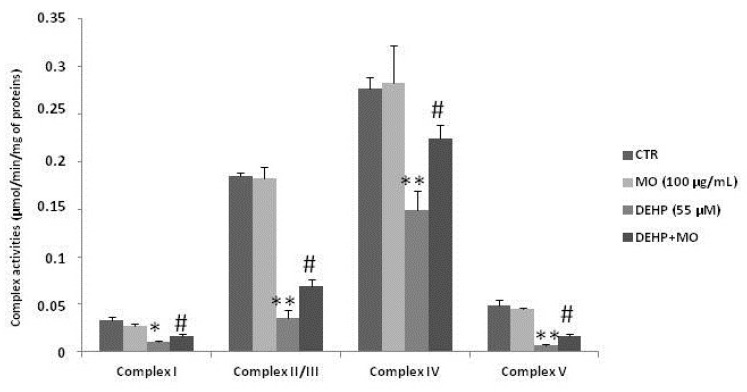
Effect of MOe on DEHP-induced mitochondrial dysfunctions. SH-SY5Y cells were pretreated with MOe (100 µg/mL) for 2 h before DEHP treatment for 24 h (55 µM). Enzymatic activities of respiratory chain complexes I, II–III, IV, and V were assayed spectrophotometrically. Data are expressed as the mean ± SD of three separate experiments. * p < 0.05, ** p < 0.01 vs. control, and *# p* < 0.05 vs. DEHP alone.

It is reported that increased ROS production after DEHP treatment can be associated with mitochondrial dysfunction, thus we analyzed the respiratory chain complex activities I, II–III, IV, and V in SH-SY5Y cells after treatment with DEHP, and then the effect of MOe pretreatment. The data illustrated in Figure 5 show that the DEHP treatment induced mitochondrial dysfunctions, as shown by a decrease in the respiratory complexes’ activities I, II–III, IV, and V. However, pretreatment with MOe reduces DEHP-induced mitochondrial alterations (*p* < 0.05).

### 3.5. Effect of MOe on NRF2 and HO-1 Expression

**Figure 6:**
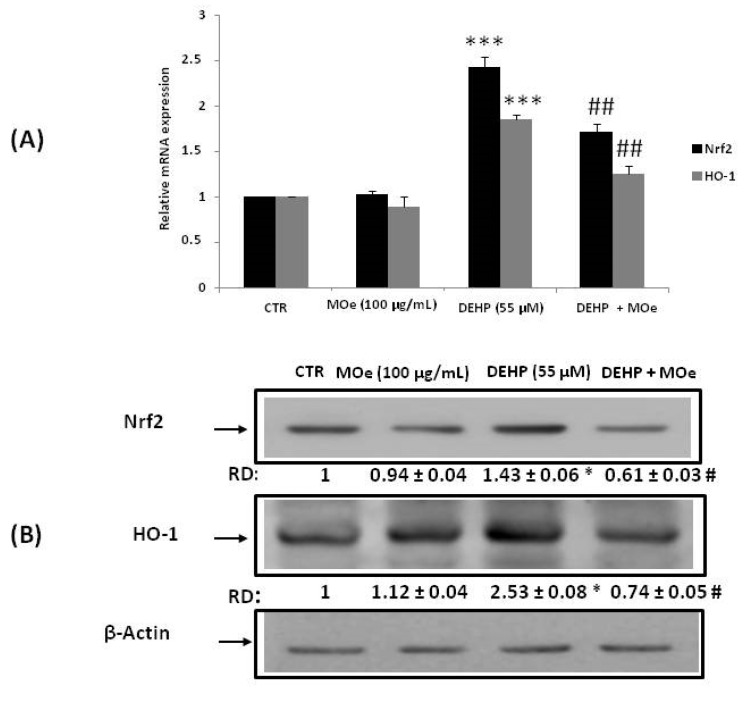
Effect of MOe on DEHP-induced upregulation of Nuclear factor erythroid 2-related factor (Nrf2) and Heme oxygenase 1 (HO-1). SH-SY5Y cells were pretreated with MOe (100 µg/mL) for 2 h before DEHP treatment for 24 h (55 µM). (**A**) Nrf2 and HO-1 relative mRNA levels were determined by quantitative Real Time (qRT)-PCR and expressed as fold change over untreated controls (**B**). As a loading control, we have used β-actin. RD: relative density as described in the Material and Methods Section.Values represent mean ± SD of three independent experiments. * p < 0.05 vs. control, *** p < 0.001 vs. control and *# p* < 0.05 vs. DEHP alone, *## p* < 0.01 vs. DEHP alone.

Under stressful conditions, the transcription factor Nrf2 is activated and leads to the transcription of different cytoprotective downstream genes, including HO-1. We estimated the gene expression of Nrf2 and HO-1 and protein levels in SH-SY5Y cells using quantitative real-time (qRT) PCR and western blot, respectively (Figure 6). Fifty-five µM of DEHP exposure demonstrated a significant increase in Nrf2 and HO-1 gene expression (Figure 6A) and protein level (Figure 6B) compared with the control group. However, pretreatment of cells with MOe for 2 h modulated their expressions by increasing the relative mRNA expression and the protein amount of NRF2 and HO-1.

### 3.6. Effect of MOe on DEHP-Induced Endoplasmic Reticulum (ER) Stress

**Figure 7:**
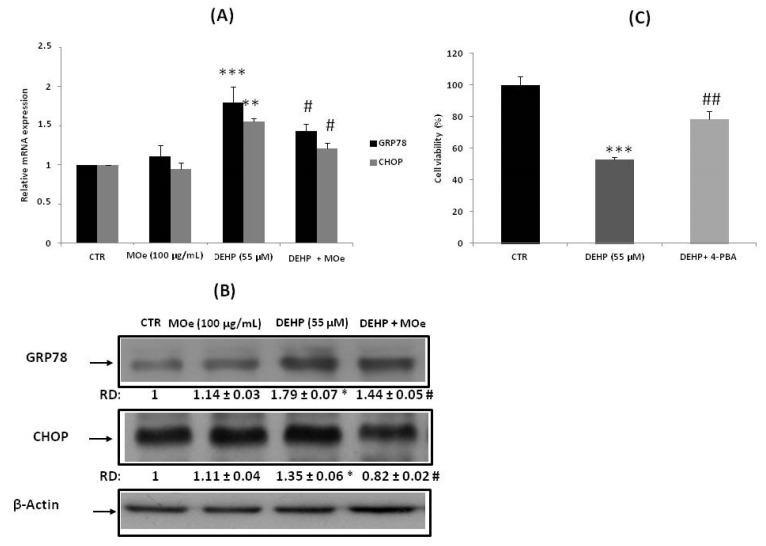
Effect of MOe on DEHP-induced endoplasmic reticulum (ER) stress. SH-SY5Y cells were pretreated with MOe (100 µg/mL) for 2 h before DEHP treatment for 24 h (55 µM). (**A**) The glucose-regulated protein 78 (GRP78) and CCAAT-enhancer-binding protein homologous protein (CHOP) mRNA levels were quantified by qRT-PCR and expressed as fold change over untreated controls. As a loading control, we have used β-actin. (**B**) GRP78 and CHOP protein levels were quantified by Western blot. RD: relative density as described in the Material and Methods Section. (**C**) Effect of 4- Phenylbutyric acid (4-PBA) pretreatment on SH-SY5Y cell viability. Values represent mean ± SD of three independent experiments. * p < 0.05, ** p < 0.01, *** p < 0.001 vs. control, and *# p* < 0.05, *## p* < 0.01 vs. DEHP alone.

We have evaluated ER stress through the expression measurement of two important markers: GRP78 and CHOP. As indicated in Figure 7A,B, exposure of cells to DEHP (55 µM) increased gene and protein expression of these proteins. However, pretreatment with the MOe (100 µg/mL) significantly decreased GRP78 expression and reduced CHOP induction (*p* < 0.05). To confirm that the DEHP induces ER stress, cells were pre-incubated for 2 h with the chemical chaperone 4-phenylbutyric acid (PBA) prior to DEHP treatment and cell mortality (MTT assay) was determined after 24 h. Consistent with the notion that PBA alleviates ER stress by stabilizing protein conformation [51,52], we observed that PBA significantly decreased cell mortality induced in response to DEHP (Figure 7C).

### 3.7. Effect of MOe on DEHP-Induced Cell Death

**Figure 8:**
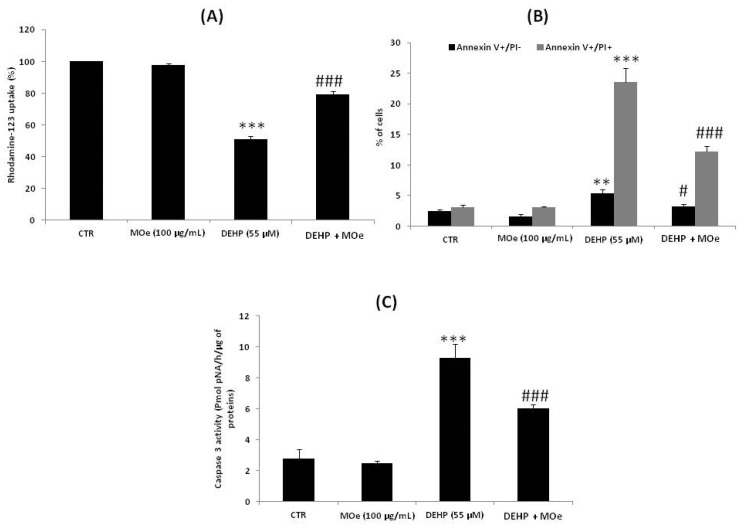
(**A**) Effect of MOe on DEHP-induced loss of mitochondrial membrane potential. SH-SY5Y cells were pretreated with MOe (100 µg/mL) for 2 h before DEHP treatment for 24 h (55 µM). After treatment, the mitochondrial potential was evaluated by the Rhodamine-123 uptake measurement. (**B**) Effects of MOe on DEHP-induced cell apoptosis. Distinct subsets of cells were detected by AnnexinV/ Propidium iodide (PI) staining after treatment with DEHP (55 µM) and/or MOe (100 µg/mL). Early apoptotic cells are positive for AnnexinV and negative for PI (AnnV+/PI−) and late apoptotic/necrotic cells are both positive for AnnexinV and PI (AnnV+/PI+). (**C**) Effect of MOe on DEHP-induced caspase-3 activation. SH-SY5Y cells were pretreated with MOe (100 µg/mL) for 2 h before DEHP treatment for 24 h (55 µM). Caspase-3 activity was measured using a commercialized kit. Data are expressed as the mean ± SD of three separate experiments. ** p < 0.01, *** p < 0.001 vs. control, and *# p* < 0.05, *### p* < 0.001 vs. DEHP alone.

On the other hand, it is known that severe ER stress induces apoptosis through the mitochondrial pathway to attenuate damage to cells [53]. Thus, we evaluated Moe’s effect on DEHP-induced mitochondrial alterations. Consistent with this purpose, Rh-123 was used to measure mitochondrial transmembrane potential (ΔΨm). As shown in Figure 8A, the percentage of Rh-123 low cells, after 24 h of DEHP exposure, reached about 49.03% ± 1.89% after treatment with DEHP. Pretreatment of cells by MOe for 2 h showed a significant decrease of Rh-123 low cells’ percentage as compared to cells treated withDEHP alone. These data demonstrate that MOe reduces the DEHP mitochondrial alterations. Moreover, double staining with PI and FITC-labeled-Annexin V allowed us to confirm the apoptosis induced by DEHP using flow cytometry. In comparison to the control values, DEHP at 55 µM increased the percentage of early apoptotic cells (AnnV+/PI-) to about 5.41% (*p* < 0.05) and late apoptotic/necrotic cells (AnnV+/PI+) to about 23.58% (Figure 8B). However, cell pretreatment with MOe for 2 h significantly reduced the apoptotic cells rate (Figure 8B). We next assessed the ability of MOe to modulate DEHP-induced caspase activation. As shown in Figure 8C, a significant (*p* < 0.05) increase in caspase-3 activity was observed in SH-SY5Y cells treated with DEHP alone, as compared to untreated cells, indicating that caspases are involved in the apoptotic process caused by the DEHP. Cells’ pretreatment with MOe, for 2 h, reduced caspase-3-activation triggered by this phthalate.

## 4. Discussion

Phthalates like DEHP remain irreplaceable as plasticizers that are used to impart flexibility and elasticity to polyvinyl chloride products. People are inevitably exposed to phthalates through environmental contamination. Exposure to DEHP has been associated with a variety of toxicological outcomes in mammals, such as reproductive toxicity, neurotoxicity carcinogenicity, hepatotoxicity, and lipid metabolism disruption.

DEHP like phthalates is used to impart elasticity and flexibility. Phthalates are irreplaceable as plasticizers to polyvinyl chloride products. Populations are inevitably exposed to phthalates through environmental contamination. Exposure to DEHP has been correlated with several toxicological outcomes in people, such as neurotoxicity, carcinogenicity, hepatotoxicity, reproductive toxicity, and lipid metabolism disruption [2,3,4,5,6]. The active compounds contained in medicinal plants have gained increasing interest in the protection of human health. In vivo and in vitro studies have demonstrated that plants or plant-derived agents can protect against DEHP-induced toxicity [54,55,56,57,58]. In this study, we investigated the beneficial effects of MOe, known for its antioxidant property, against DEHP-induced toxicity in SH-SY5Y cells. We showed that SH-SY5Y cells are susceptible to DEHP exposure, which results in a decrease in the cell viability compared to the untreated cells. Moreover, we demonstrated that 2 h pretreatment with MOe significantly increased cell viability altered by the DEHP. Our results also report that treatment of SH-SY5Y cells with DEHP for 24 h induced overproduction of ROS. These ROS can cause cell damage, resulting in cell death. For this reason, the use of antioxidant molecules and radical scavengers can plausibly prevent cell damage [59]. In fact, pretreatment with MOe (100 µg/mL) significantly protected cells against DEHP-induced oxidative stress by decreasing the ROS production. This result is in agreement with published data demonstrating that MOewas discovered to prevent but also protect against the oxidative stress and cognitive disordersin hyperhomocysteinemia-induced Alzheimer’s Disease-like pathology in rats [60]. In addition, it has been recently demonstrated that MOeattenuated titanium oxide nanoparticles-induced brain damage in rats by restraining oxidative stress [20]. This preventive effect against ROS-induced cell damage has also been highlighted in other cellular systems and can therefore be considered a generalized effect.In fact, published data report that MOe protected human neural cells from hydrogen peroxide (H_2_O_2_)-induced oxidative stress [61]. In addition, it has recently been demonstrated that MO attenuates the high glucose-induced ROS overproduction in vascular smooth muscle cells (VSMCs) [62], and also, MOe exhibits protection of skin keratinocytes against oxidative stress, by activation of the antioxidant defenses and Peroxisome proliferator-activated receptor alpha (PPARα) [63]. Consistent with this finding, MOe exerted a protective role on antioxidant status in heart muscle and lipid peroxidation in streptozocin-induced diabetic models in rats [64]. The observed antioxidant effect of MO is comparable to that tested by other antioxidant molecules: alpha lipoic acid, melatonin, lycopene, and silymarin, that have shown a protective effect against oxidative stress induced by DEHP [65,66,67,68].

The increased ROS production can be associated with mitochondrial dysfunction and impairment of mitochondrial electron transport chain function [69,70]. Our results clearly show that DEHP treatment of SH-SY5Y is associated with mitochondrial dysfunction, as revealed by decreased activity respiratory complex I, II–III, IV, and V, and mitochondrial membrane potential. DEHP-induced mitochondrial dysfunction could be associated with several mitochondrial deregulations, such as biogenesis, reduced number of viable mitochondria, and mitochondrial fusion/fission. In addition, the alterations in respiratory complex activities could result from the redoximbalance, which causes degeneration in cells [71].However, further studies are necessary, to define whether DEHP can alsodetermine post-translational and structural modifications of these cellular enzymes. Oxidative stress plays a key role in mitochondrial dysfunctions and could be associated with oxidative modifications of subunits of respiratory chain complexes and modulation/deregulation of their activities [71,72,73]. On the other hand, our data show that MOe pretreatment could preserve mitochondrial dysfunction, as shown by the effect of MOe on the mitochondrial complexes I, II–III, IV, and V, supporting a key role in keeping mitochondrial function, thus renewing the capacity of neurons to produce energy.

The increase of ROS generation is expected to trigger an array of antioxidant systems to maintain cellular homeostasis. Nrf2 is an inducible transcriptional factor upregulating most antioxidant enzymes. Nrf2 is an essential inducer of defense enzymes in cellsand to involve many genes participating in stress-related processes including anti-inflammation, antioxidation, detoxification, metabolism, differentiation, and cell proliferation [74]. To counteract the damage insults, mammalian cells have evolved signaling mechanisms to turn off or on antioxidant responses and cellular adjustments. A well-known endogenous antioxidant response inducedthe activation ofantioxidative and detoxification enzymes through the system Nrf2-Keap1. Under normal conditions, Keap1 stops the Nrf2 factor within the cytoplasm to decrease the levels of Nrf2 that check the constitutive expression of Nrf2 downstream genes. In cells exposed to oxidative imbalance, a signal implying redox modification of critical cysteine residues in Keap1 blocks ubiquitination and proteasomal degradation. In the nucleus, the free Nrf2 translocates and transactivates the antioxidant response elements (AREs) of several cytoprotective genes [75].Thus, cellular expression of Nrf2 is necessary to identify the susceptibility to oxidative stress. Upregulation of Nrf2 and its target protein HO-1, studied in thisresearch, might be due to the production of oxidative stress created by the DEHP, which, on the other hand, was modulated by MOe pretreatment. Theseresults support the notion that MOe is endowed with neuroprotective activity and is in agreement with another study reporting that MOe alleviated titanium oxide nanoparticles-induced neurotoxicity in rats, as revealed by significant upregulation of Nrf2 and NQO1 mRNA expression [20].

Several evidences have demonstrated that, in *Caenorhabditis elegans*, the inducible transcription factor skinhead-1 (SKN-1), a homologue of mammalian Nrf proteins, directly controls the ER unfolded protein response (UPR) signaling and controls the transcription factor genes of XBP-1 and ATF-6 [76]. Moreover, responses to oxidative stress mediated by SKN/Nrf were dependenton ER signaling. SKN-1/Nrf was established aspivotal for resistance to ER stress [77]. For this reason, we looked for the implication of ER stress after studying the response of Nrf2.

In the case of exogenous or endogenous stress, the cell could undergo ER stress that activates the UPR. Under ER stress conditions, the cell activates different pathways, Activating transcription factor 6 (ATF-6), and Inositol-requiring enzyme 1(IRE-1), in order to stimulate protein folding, via Binding Immunoglobulin Protein (BiP) induction, and thus decreasing the accumulation of misfolded proteins [78]. Recent studies have confirmed that Nrf2 is also an important downstream target of PERK. PERK phosphorylation activates Nrf2 nuclear translocation [79].

In this study, we demonstrated the involvement of the PERK branch given that the phosphorylation of PERK can activate the Nrf2 pathway [79]. Moreover, it has been observed that extract of MO leaves exerts a beneficial effect on *Caenorhabditis elegans*, prolonging the lifespan via activation of DAF-16 (Fork-head domain-containing protein-16), AGE-1 (Phosphatidylinositol 3-kinase age-1), SIR-2.1 (Deacetylase sirtuin-type domain-containing protein;NAD-dependent protein deacetylase sir-2.1), SKN-1 (Protein skinhead-1), and DAF-2 (Insulin-like receptor subunit beta) [80]. Our data showed that treatment with DEHP induced ER stress, as proven by an increase in the expression of the chaperone CHOP and GRP78 mRNA. To better ensure that ER stress is activated by DEHP, we pretreated the cells with the ER stress inhibitor, 4-PBA, 2 h before exposure to DEHP, and then we tested the cell viability using the MTT assay. Our results prove that ER stress inhibition increased cell viability compared to cells not pretreated with 4-PBA. Different reports have suggested that treatment with DEHP also induces ER stress in human immortalized keratinocyte (HaCaT) cells as well as in mouse testes [81]. In parallel, we observed that 2 h pretreatment of cells by MOe decreased protein expression of GRP78 and CHOP, indicating that MOe can attenuate the ER stress caused by the DEHP. It was also elucidated that MOe decreased the ER stress in high-fat diet-induced obese mice [82]. In severe stress, the UPR directs the cell to death by activating the apoptotic pathway. The CHOP was the transcription factor recognized as a key mediator of ER stress-induced apoptosis and is upregulated by the three UPR branches [83]. An excess of ROS production can induce ER stress and initiate the mitochondrial pathway of apoptosis [84]. The inhibition of the mitochondrial enzyme complexes’ activities is an upstream event for the reduction of the mitochondrial membrane potential. An overall decrease in the mitochondrial membrane potential acts as a potent mediator in cell death [85,86]. This potential can be reduced in moderate cell damage and enlarge the mitochondrial pores of the outer membrane. This leads to the cytochrome c release into the cytoplasm and triggers apoptosis [84]. Our results indicate that DEHP induces an ΔΨm dissipation, which can cause a permeabilization of the mitochondrial membrane, and activates a proteolytic enzyme, the caspase-3, which is the major cell death effector protease and is an indicator of apoptotic phenotypes [87].These data suggest that ER stress caused by the DEHP could be associated with the induction of apoptosis via the caspase-dependent mitochondrial pathway. Moreover, our evidence suggests that MOe may prevent DEHP-induced mitochondrial damage by stabilizing membrane potential and decreasing caspase activation in the mitochondrion. A recent paper demonstrated that pretreating human neuron cells with MOe showed a significant resistance to H_2_O_2_-induced apoptotic cell death [88].

## 5. Conclusions

For the first time, in the present work, we reported that MOe exerts neuroprotective effects against DEHP-induced cytotoxicity in SH-SY5Y cells. By reducing ROS production, MOeprotects cells and inhibits the ER stress response from DEHP-induced mitochondrial apoptosis. Increasing interest is emerging on the development of functional compounds capable to modulate cytoprotection and thus provide health-promoting effects. Our results demonstrated the effectiveness of novel approaches in preventing DEHP-induced cell damage and more in general to pursue health-promoting outcomes.

## Figures and Tables

**Table 1:** List of identified compounds and accurate masses.

GC-MS/MS Analysis		
Retention Time (minutes)	Name	Relative abundance (%)
10.39	Astaxanthin	0.91
12.7	Carotene, 1,1′,2,2′-tetrahydro-1,1′-dimethoxy	0.18
13.46	1-Monolinoleoylglycerol	1.01
17.3	Decanoic acid	0.19
17.78	Rhodopin	3.05
19.41	Destruxin A	0.19
20.46	Hexadecanoic acid, ethyl ester	0.18
20.79	5,8,11,14,17-Eicosapentaenoic acid, methyl ester,	1.05
22.07	9-Octadecynoic acid, methyl ester	2.01
22.18	Methyl 8,11,14-heptadecatrienoate	1.09
27.64	2-Nonadecanone 2,4-dinitrophenylhydrazine	3.49
28.82	Quercetin	5.31
30.08	Gallic acid	41.8
32.47	Oleic acid, 3-(octadecyloxy)propyl ester	13.26
35.14	Vitamin E	6.91
36.25	Kaempferol	7.79
37.21	β-Sitosterol	6.63
39.12	Oleyl oleate	4.95
**LC-Orbitrap^TM^-MS Analysis**		
Retention Time (minutes)	Name (accurate masses)	Relative abundance (%)
2.71	Gallic acid (168.96)	11.45
3.61	Chlorogenic acid (353.09)	11.21
5.02	Vanillin (150.99)	4.52
5.72	Syringic acid (197.10)	10.63
6.66	Ferulic acid (193.03)	14.75
6.84	Rutin (609.11)	3.32
7.32	Quercetin (301.01)	15.11
8.53	Kaempferol (285.04)	29.01

## Data Availability

All data are presented in the paper.

## Abbreviations

MO	Moringa oleifera
SH-SY5Y	Human neuroblastoma cells
DEHP	Di-(2-ethylhexyl) phthalate
ROS	Reactive oxygen species
ER	Endoplasmic reticulum
Nrf2	Nuclear factor erythroid 2-related factor
Keap1	Kelch-like ECH-associated protein 1
ARE	Antioxidant response element
NQO1	NAD(P)H:quinone oxidoreductase 1
HO-1	Heme oxygenase 1
GST	Glutathione S-transferase
HSP	Heat shock proteins
PC12	Pheochromocytoma 12 cells
PBA	Chaperone 4-phenylbutyric acid
ΔΨm	Mitochondrial transmembrane potential
VSMCs	Vascular smooth muscle cells
Complex I	NADH:ubiquinone oxidoreductase
Complex II-III	Succinate-cytochrome c oxidoreductase
Complex IV	Cytochrome c oxidase
Complex V	ATP synthase
CTR	Control
UPR	Unfolded protein response
DMEM	Dulbecco’s Modified Eagles Medium
FBS	Fetal Bovine Serum
PBS	Phosphate Buffer Saline
DCFH-DA	2,7-dichlorodihydrofluorescein diacetate
MTT	3-4,5-Dimethylthiazol-2-yl,2,5-Diphenyltetrazolium Bromide
DCFH	Non-fluorescent dichlorodihydrofluorescein
AnnV/PI	Annexin V/propidium iodide
AMC	Aminomethylcoumarin
